# Evaluation of the Development, Implementation, Maintenance, and Impact of 3 Digital Surveillance Tools Deployed in Malawi During the COVID-19 Pandemic: Protocol for a Modified Delphi Expert Consensus Study

**DOI:** 10.2196/58389

**Published:** 2024-12-31

**Authors:** Alanna Denny, Isaach Ndemera, Kingston Chirwa, Joseph Tsung Shu Wu, Griphin Baxter Chirambo, Simeon Yosefe, Ben Chilima, Matthew Kagoli, Hsin-yi Lee, Kwong Leung Joseph Yu, John O'Donoghue

**Affiliations:** 1 Research Department Luke International Mzuzu Malawi; 2 School of Public Health Department of Medicine & Health University College Cork Cork Ireland; 3 Research Department Mzuzu University Mzuzu Malawi; 4 Department of Overseas Mission Pingtung Christian Hospital Pingtung Taiwan; 5 Nursing and Midwifery Department Faculty of Health Sciences Mzuzu University Mzuzu Malawi; 6 National Statistics Office Zomba Malawi; 7 Public Health Institute of Malawi Lilongwe Malawi; 8 Research Department Luke International Nøtterøy Norway; 9 Business Information Systems University College Cork Cork Ireland; 10 ASSERT Research Centre University College Cork Cork Ireland; 11 Malawi eHealth Research Centre Mzuzu University Mzuzu Malawi

**Keywords:** delphi study, COVID-19, Malawi, surveillance, digital health, delphi, mobile health, mHealth, development, implementation, maintenance, impact, consensus, protocol, survey, expert, purposive sampling, epidemiology, descriptive statistics

## Abstract

**Background:**

The COVID-19 pandemic has highlighted the importance of strengthening national monitoring systems to safeguard a globally connected society, especially those in low- and middle-income countries. Africa’s rapid adoption of digital technological interventions created a new frontier of digital advancement during crises or pandemics. The use of digital tools for disease surveillance can assist with rapid outbreak identification and response, handling duties such as diagnosis, testing, contact tracing, and risk communication. Malawi was one of the first countries in the region to launch a government-led coordinated effort to harmonize and streamline the necessary COVID-19 digital health implementation through an integrated system architecture.

**Objective:**

The aim of this study is to seek expert consensus using the Delphi methodology to examine Malawi’s COVID-19 digital surveillance response strategy and to assess the digital tools using the World Health Organization mHealth (mobile health) Assessment and Planning for Scale (MAPS) toolkit.

**Methods:**

This protocol follows the Guidance on Conducting and REporting DElphi Studies. Participants must have first-hand experience on the design, implementation or maintenance with COVID-19 digital surveillance systems. There will be no restrictions on the level of expertise or years of experience. The panel will consist of approximately 40 participants. We will use a modified Delphi process whereby rounds 1 and 2 will be hosted online by Qualtrics and round 3 will encompass a face-to-face workshop held in Malawi. Consensus will be defined as ≥70% of participants strongly disagree, disagree, or somewhat disagree, or strongly agree, agree, or somewhat agree. During round 3, the face-to-face workshop, participants will be asked to complete, the MAPS toolkit assessment on the digital tool on which they are experts. The MAPS toolkit will enable the panel members to assess the digital tools from a sustainable perspective from six distinct, yet complementary axes: (1) groundwork, (2) partnerships, (3) financial health, (4) technology and architecture, (5) operations, and (6) monitoring and evaluation.

**Results:**

The ability of a country to collate, diagnose, monitor, and analyze data forms the cornerstone of an efficient surveillance system, allowing countries to plan and implement appropriate control actions. Malawi was one of the first countries in the African region to launch a government-led coordinated effort to harmonize and streamline the necessary COVID-19 digital health implementation through an integrated system architecture.

**Conclusions:**

We anticipate findings from this Delphi study will provide insights into how and why Malawi was successful in deploying digital surveillance systems. In addition, findings should produce recommendations and guidance for the rapid development, implementation, maintenance, and impact of digital surveillance tools during a health crisis.

**International Registered Report Identifier (IRRID):**

DERR1-10.2196/58389

## Introduction

### Background

Emergent zoonotic diseases have had disastrous global consequences, particularly in low- and middle-income countries (LMICs). LMICs are also impacted strongly by endemic and overlooked zoonotic illnesses [1]. Despite investments by the global health community, in increasing disease surveillance systems capacities across LMICs, initiatives need to be more cohesive and frequently fail to reach communities in remote rural areas [2]. Insufficient surveillance in such communities can lead to disease outbreaks being detected at a later stage and responded to ineffectively, thus, increasing the likelihood of pandemics inflicting greater damage due to latent amplification along with regional and global travel. The COVID-19 pandemic has highlighted the importance of strengthening national monitoring systems in order to safeguard a globally connected society, especially those in LMICs [3]. To combat the COVID-19 pandemic, local governments, nongovernmental organizations, and individuals have reused existing technologies and developed innovative solutions [4].

A 2022 study that conducted thematic synthesis of the World Health Organization intra-action review reports in 18 African countries noted that findings were suggestive that African countries responded quickly to the virus and should be commended for their efforts [5]. In particular, Africa’s rapid adoption of digital technological interventions created a new frontier of digital advancement during crises or pandemics [6]. The use of digital tools for disease surveillance can assist with rapid outbreak identification and response, handling duties such as diagnosis, testing, contact tracing, and risk communication [6].

During the COVID-19 pandemic, the dominant type of digital surveillance in Africa was track and trace systems, which were largely obtained from telecommunications data and specific apps. However, during national lockdowns, nations such as Ethiopia and Sierra Leone implemented internet-based payment systems and electronic travel pass management to ensure distancing amongst travelers and to restrict movement to vital personnel [7]. Drones were used in Morocco, Sierra Leone, and Tunisia to send warnings and notifications, as well as to ask individuals on the street to provide their motives for being outdoors during lockdowns [8-10]. Furthermore, technologies that enable mass communication were used to disseminate messages relating to prevention measures. For example, a Moroccan startup launched a software based artificial intelligence bot that answered COVID-19–related inquiries in Arabic [11]. In addition, to preventative measures, multiple African nations implemented systems (such as national disease surveillance dashboards) to track the number of cases, testing, and deaths. Ethiopia designed smartphone apps for capturing personal identification data and temperatures at ports of entry, which were then used by a COVID-19 surveillance system for contact tracing [12]. Active community participation is required for the effectiveness of public health programs such as social distancing and mask-wearing. Early community engagement with technology and advances during the pandemic resulted in numerous contextualized approaches.

The COVID-19 pandemic had an immense impact worldwide, exposing significant flaws and vulnerabilities in our health care systems. Furthermore, the COVID-19 pandemic highlighted the significance of robust and well-functioning surveillance systems, given their role in local decision-making and worldwide knowledge exchange. The ability of a country to collect, collate, diagnose, monitor, and analyze data forms the cornerstone of an efficient surveillance system, allowing countries to plan and implement appropriate control actions. Malawi was one of the first countries in the region to launch a government-led coordinated effort to harmonize and streamline the necessary COVID-19 digital health implementation through an integrated system architecture.

In its Monitoring, Evaluation, and Health Information Systems Strategy, Malawi’s Ministry of Health emphasized strengthening health information systems. Its primary goals were to establish interoperable digital systems that are loaded with high-quality data to promote data use in decision-making. The COVID-19 pandemic heightened the importance of this goal. It was a straightforward and economical move to use digital health tools to reinforce the Monitoring, Evaluation, and Health Information Systems Strategy objectives and expedite Malawi’s COVID-19 response. Malawi’s health system uses 54 digital health instruments, at least 20 of which have already been deployed for COVID-19 pandemic (Multimedia Appendix 1). The Ministry of Health Digital Health Division and the Public Health Institute of Malawi, with the assistance of Luke International and other development partners, rapidly created and deployed a suite of digital tools that aided the country in its COVID-19 response. This includes the One Health Surveillance Platform (OHSP), which is based on District Health Information Software 2 (University of Oslo). The OHSP was adopted as the single repository for COVID-19 interventions, including data for port of entry screening, case-based surveillance, contact tracing, confirmed case management and investigation, COVID-19 school assessment, COVID-19 e-Vaccine registry, and Integrated Disease Surveillance and Response reports (Multimedia Appendix 1). Furthermore, Luke International and its partners developed the Ministry of Health’s COVID-19 National Information Dashboard, granting individuals with internet access the opportunity to review statistics in Malawi [13].

### Goal of The Study

In this paper, we will seek expert consensus using the Delphi methodology to examine Malawi’s COVID-19 digital surveillance response strategy, that is, the use of the following 3 digital tools.

#### Integrated Disease Surveillance and Response Case-Based Surveillance App Malawi

This application extends the existing infrastructure of electronic medical records and implements an electronic integrated disease surveillance and response system to facilitate real-time detection and reporting of notifiable disease cases. The application sends text alerts to all people under quarantine and thus, allows for the monitoring of people under quarantine.

#### Emergency Operations Center Call Center Exchange

The Emergency Operations Center (EOC) Call Center Exchange is a distributed call management system. It uses private automatic branch exchange for a more effective way of communication. The system improves the technical capacity to intelligently handle and manage calls, directing them to respective districts where they will be attended to by dedicated and trained district call center agents. This tool enhances the EOC to a modern solution that decentralizes the management of phone calls from the general public regarding COVID-19 pandemic.

#### One Health Surveillance Platform (by District Health Information System 2)

OHSP records and reports disease surveillance data in a holistic approach that involves human, animal (livestock and wild), and environmental aspects to disease surveillance. This web and mobile platform allows for patient screening, patient tracking and follow-up, contact tracing, case management, vaccine delivery and planning, and laboratory sample tracking.

Finally, we plan to assess the digital tools using the World Health Organization mHealth Assessment and Planning for Scale (MAPS) toolkit [14]. The MAPS toolkit is a robust “self-assessment and planning guide” that aims to advance conversations on how to scale up and maximize the impact of mobile health (mHealth) innovations [14]. Furthermore, the toolkit aims to optimize strategies to attain long-term sustainability.

## Methods

### Overview

This study will follow the Guidance on Conducting and REporting DElphi Studies [15].

### Study Design

This study will use a modified Delphi technique that repeats a process of gathering and refining expert opinions until consensus is obtained. It is based on the concept that the opinions and predictions of an ensemble of experts are more reliable or accurate than those provided by individual experts [16]. The Delphi technique was chosen as the most appropriate study methodology to establish explicit consensus-based criteria where there is inadequate quantity or quality of existing evidence to develop evidence-based criteria [17]. Furthermore, the Delphi method is an iterative process technique that brings together the insights of various experts to establish a consensus and is used to generate future predictions, devise policy alternatives and find solutions. Our topic of study is under-research and so lacks existing evidence.

In the traditional Delphi process, there is no face-to-face interaction between the expert panel. However, we will undertake a modified Delphi process, in which round 3 will encompass a face-to-face workshop with the expert panel an approach which has been taken previously [18,19]. Notably, only those who completed both rounds 1 and 2 will be eligible to take part in the face-to-face workshop as these are the experts who can best speak on recommendations and feedback to the statements. This face-to-face workshop with experts who took part in the previous 2 rounds will facilitate discussion of the statements and recommendations in real-time by the experts, which will improve the efficiency of the consensus process whilst also maintaining anonymous voting [20]. During the group discussions of statements that did not reach consensus, experts will be advised not to reveal how they voted in the previous rounds. Furthermore, after finalization of the list of round 3 statements, experts will be asked to rerate the statements individually online using Qualtrics (Silver Lake) survey software to maintain anonymity amongst participants.

Experts who took part in rounds 1 and round 2 but are unable to attend the face-to-face workshop will be sent an email that asks them to provide feedback and recommendations to the statements, before the workshop. Any feedback provided by email from experts who are eligible but unavailable for workshop will be discussed and presented during the workshop. This process will ensure that all who are eligible to provide recommendations and feedback to the Delphi statements have an opportunity to do so.

It should be noted that experts who did not complete round 1 and round 2 will not be eligible to participate in round 3. However, experts that have completed either round 1 or round 2 but not both rounds will be eligible to participate in round 3 of consensus but this will be through Qualtrics and not in person at the face-to-face workshop.

Finally, if there is no response to the Qualtrics survey, reminder emails, phone calls, text messages may be used to try to enhance response rates but ultimately with any research study, the response rate is based on the discretion of the respondent [21].

### Study Setting

**Figure 1:**
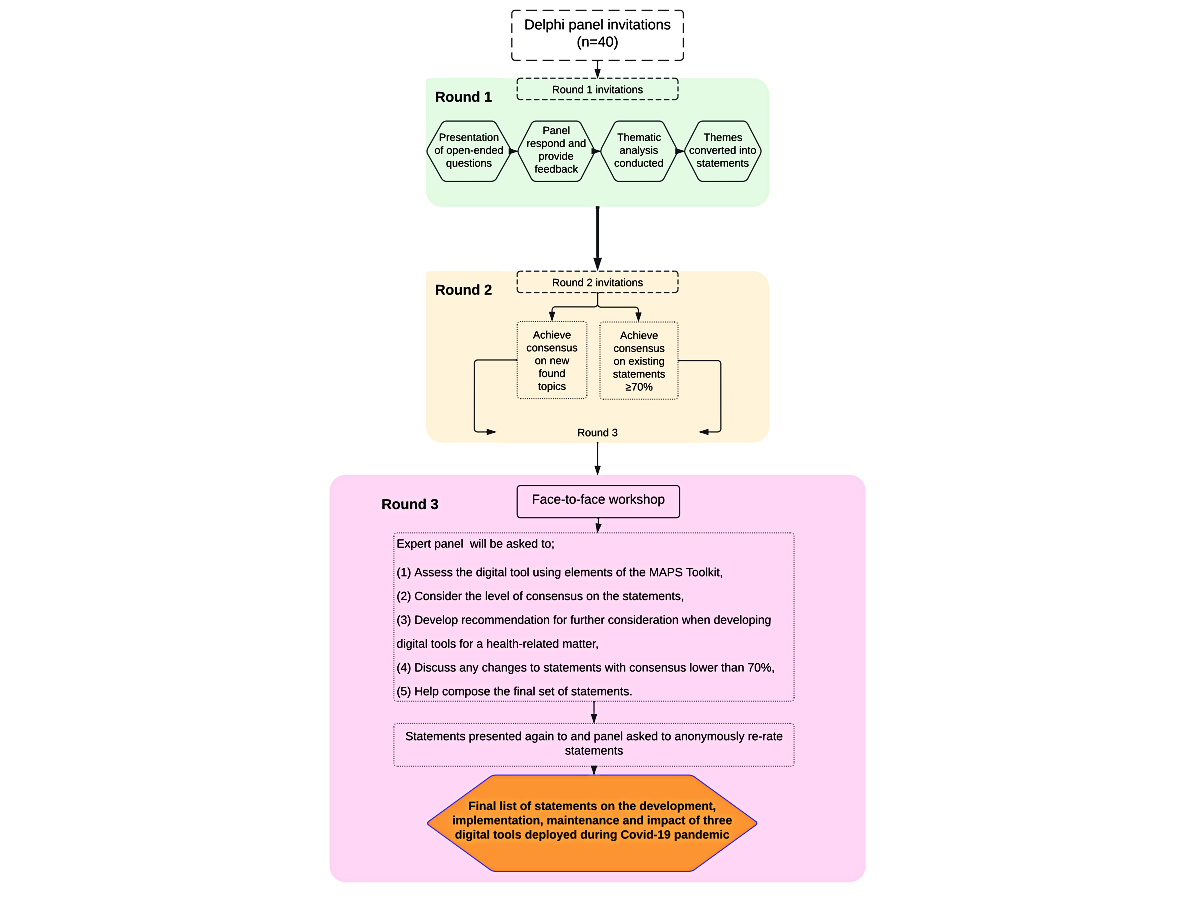
Delphi study flow diagram.

Delphi study rounds 1 and 2 will be conducted online through Qualtrics, a survey platform [20]. The Delphi study round 3 face-to-face workshop will be held at a site and location in Malawi convenient to the participants (Figure 1). This will be decided after recruitment and identification of consenting individuals in order to find a central location for all experts. Experts who are not eligible or unable to attend the face-to-face workshop will be able to rate the statements online through Qualtrics.

### Study Participants

For this study, the participants will be referred to as an expert panel or panel of experts. In a Delphi study, there must be heterogeneity among the panel of experts. Participants must have first-hand experience (design, implementation, use, or maintenance) with COVID-19 digital surveillance systems. There will be no restrictions on the level of expertise or years of experience in their field of work.

The panel will consist of approximately 40 participants (Textbox 1).

Participants will be excluded if they lack the necessary expertise or are unable to make a commitment to being available for the duration of Delphi study rounds. If potential stakeholders are unable or decline to participate in the study, they will be invited to recommend a suitable alternative from their field.

**Recruitment sources for study participants.**

**Ministry of Health (the below stakeholders report back to the Ministry of Health)**

- Digital Health Division.
- Public Health Institute of Malawi.
- Border health officials.
- Integrated disease surveillance and response stakeholders.

**Implementing entities**

- District Health Offices.
- Central Monitoring and Evaluation Division.
- Non-governmental organizations: Elizabeth Glaser Pediatric AIDS Foundation, Luke International, Angle Dimension, and VillageReach.

### Sampling Methods

**Figure 2:**
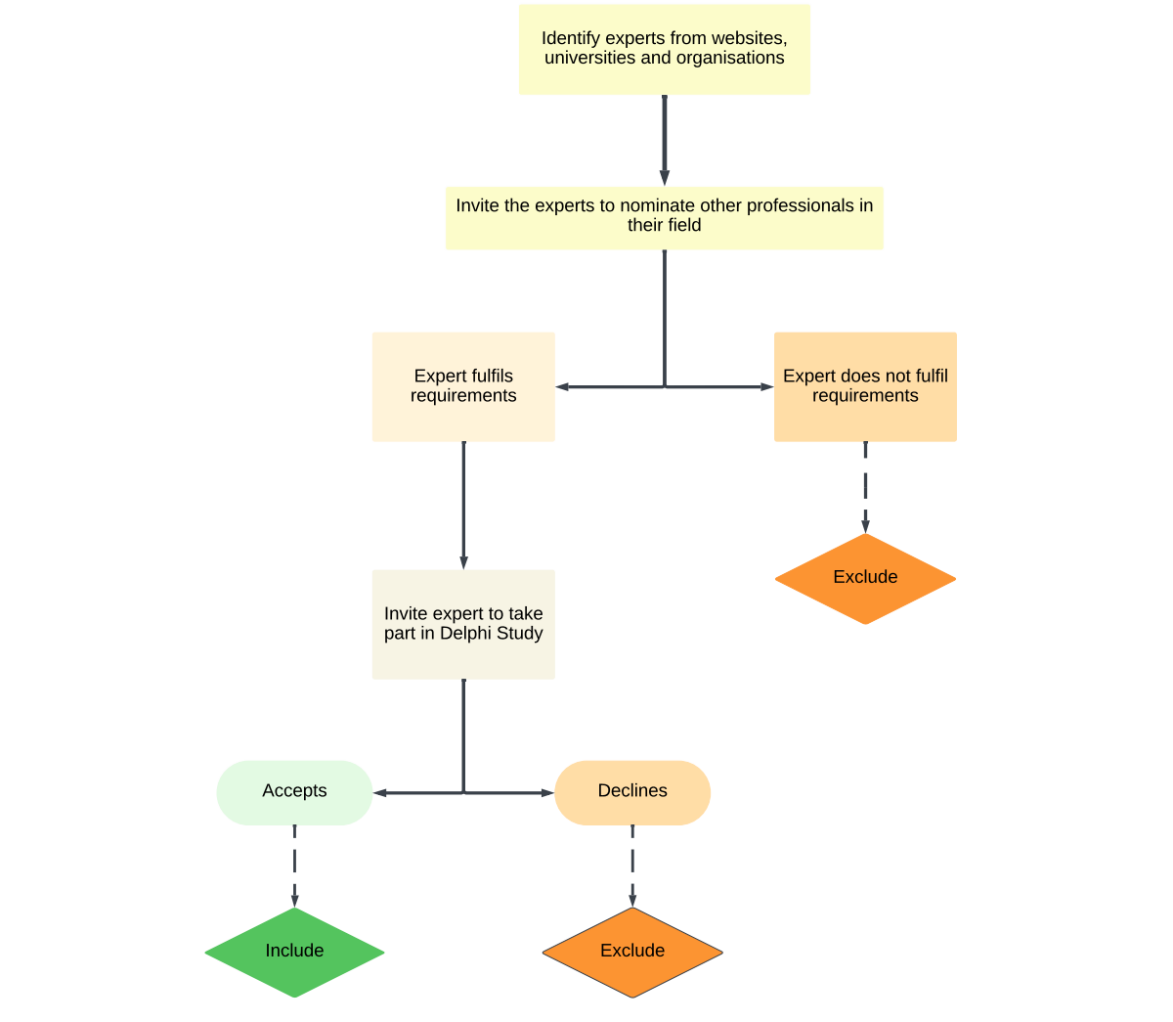
Recruitment strategy.

Nonprobability purposive sampling will be used to invite participants (Figure 2). Purposive sampling techniques are used in each strategy to identify people who have first-hand knowledge of the phenomenon being studied, and as Patton [22] notes, it is the selection of “information-rich cases.” Through peer recommendations and letters to appropriate professional groups, we will find relevant experts. We will get in touch with these organizations to obtain permission to send letters of invitation to their members. Snowball sampling will be implemented, which allows participants to recommend other persons with knowledge of COVID-19 digital systems [23].

### Sample Size

There is no established standard for panel sample size, however it is widely agreed that the greater the number of participants, the greater the reliability of group judgements. Delphi technique panels range in number from 10 to over 1000, and there is no consensus or set of criteria about the appropriate size in the literature [24,25]. As there is no formal sample size calculation in Delphi studies, the literature was reviewed, in order to determine the most common and appropriate sample size whilst ensuring sufficient representation of the population. A 1995 study noted that the approximate size of a Delphi panel is generally under 50 and previous studies advise a panel of 40 as an overall rule [26,27]. Based on previous literature, the research team agreed on 40 experts to be included.

### Recruitment

Representatives from each of the aforementioned stakeholder groups will be invited to participate in the Delphi study. We will email each of the stakeholders and provide an information sheet and consent form.

### Information Sources and Steering Committee

For this study, the steering committee are the experts who will assist in the design and development of questions and statements for the Delphi rounds.

As this is a niche area with limited research, we did not conduct a formal literature search commonly seen in the Delphi process. Instead, the first step to preparing our Delphi study was to identify key terms relating to digital surveillance tools for COVID-19 pandemic. To do so, we engaged with the research team, which is comprised of persons who have or have previously worked in one of our stakeholder groups, have knowledge of digital surveillance tools, or are experienced in Delphi study design (Table 1).

**Table 1:** Delphi study steering committee members expertise.

Name (anonymized to maintain confidentiality of the committee)	Expertise
Member 1	Epidemiology Surveillance Emergency response Digital health development
Member 2	Statistics
Member 3	Public health Epidemiology
Member 4	Public health Microbiology
Member 5	Software developer Digital health
Member 6	Qualitative expertise
Member 7	Qualitative expertise Community health

The panel is composed of 4 members of the research team and 3 independent experts. These independent experts were recruited with the sole purpose of assisting with the development of the Delphi questions and statements. The experts were informed of the purpose of the study, the anticipated time they would be required to dedicate to the study and that their involvement would be voluntary. Meetings on Zoom (Zoom Video Communications), WhatsApp (Meta), and email were used to design and review feedback on the questions. In addition, the MAPS toolkit was used to help form the questions.

Overall, the steering committee will work collaboratively to reach an agreement on the Delphi survey format, statements, consensus thresholds, and stakeholder selection.

### Data Collection

A Delphi study collects input from multiple expert panelists through a series of questionnaires or statements. In contrast to other data-gathering strategies, Delphi studies use processes of feedback (iterations) to build a consensus on a given topic. Given that the number of rounds in a Delphi can vary, there will come a time when additional agreement or consensus is either unnecessary or improbable to be obtained [28]. Whilst there has been no guideline on what constitutes an acceptable response rate, we do anticipate there will be a drop-off in the number of participants. The COMET handbook estimates that an appropriate response rate for each stakeholder group is around 80% [28]. The research team will endeavor to have a robust recruitment strategy to achieve this. However, for our study, as it is a niche area of expertise, response rate will not be a concern for reaching consensus and we will continue to launch subsequent rounds if the response rate is poor. With that said, we will report both the level of consensus and the response rate for each round so as to be as transparent as possible about our findings.

Reminder emails, phone calls, text messages may be used to try to enhance response rates but ultimately with any research study, the response rate is based on the discretion of the respondent [21].

### Consensus Definition

There are several approaches proposed to define the consensus criteria [28]. Consensus will be defined as ≥70% of participants strongly disagree, disagree, or somewhat disagree or strongly agree, agree, or somewhat agree [29]. 

As we are conducting a Delphi study on 3 digital tools, we will create 3 separate forms on Qualtrics for each of these tools. Stakeholders will be instructed only to complete the survey for the digital tool that they are experts on, and to ensure this, the experts will be emailed an anonymous link only for their designated digital tool. An information sheet will accompany each Delphi Round on the aims and procedures of this Delphi Study. The approach taken for this Delphi is the “all-rounds” approach whereby participants who do not respond to one are still invited to respond to others [30].

The decision to stop the Delphi process for a given topic will be based on reaching the consensus threshold or completion of 3 rounds (Figure 1).

### Stage 1: First Round

#### Overview

Participants will receive an anonymous link to complete round 1 on Qualtrics. Round 1 will comprise a list of open-ended questions on the development, implementation, maintenance, and impact of each of the aforementioned digital tools that were designed by the steering committee. This is in line with traditional Delphi studies, whereby the initial round of a Delphi study starts with an open-ended questionnaire designed to elicit detailed information on the topic from the panel of experts [17]. Participants will be asked to add a comment, rationale, or suggestion for rewording if needed. Experts can also add free text suggestions regarding the design, implementation, maintenance, and impact of the digital tool.

#### Data Analysis

Responses to open-ended questions from the questionnaire will be summarized qualitatively using thematic analysis. Thematic analysis is a qualitative method that is used to identify, analyze, and report patterns or themes found within data [31]. For this Delphi study, we will adopt steps 1-6 of Braun and Clarke’s guide to thematic analysis [31]. Data from Qualtrics will be extracted verbatim onto a Google Spreadsheet. One author (AD) will familiarize themselves with the data and generate the initial codes. Afterwards, we will create themes, review, and refine (research team). Furthermore, feedback from round 1 will be discussed by the study team and steering committee for potential incorporation into round 2.

### Stage 2: Second Round

#### Overview

Themes identified from participant responses in round 1 will be used to generate statements for round 2 [30]. Round 2 will consist of a traditional structured questionnaire composed of statements hosted on Qualtrics. Participants will be presented with a 7-point Likert scale for each statement with the following options: strongly disagree, disagree, somewhat disagree, either agree or disagree, somewhat agree, and agree. There is an opportunity after each statement for additional remarks, and any statements with a consensus level less than the recommended threshold of ≥70% will be explored further in round 3 [32]. Statements with a consensus level less than the recommended threshold of ≥70% will be brought to the steering committee for review along with any feedback given by the panel. Participants will be asked to provide reasons for their disagreement with the statements if any.

#### Data Analysis

Central tendencies (means and medians) and levels of dispersion (IQR) will be computed from round 2 to present to participants in round 3.

### Stage 3: Third Round (Face-to-Face Consensus Workshop)

#### Overview

Before round 3, results from round 2 will be summarized and circulated to the panel members, providing a summary of the level of group agreement (percentage of those who agreed or strongly agreed) and a summary of the submitted comments and suggested rewording of statements from round 2. Only the survey statements that do not achieve consensus from round 2 will be presented in round 3. Experts will be advised not to discuss how they rated the statements so to maintain anonymity for the previous rounds. The face-to-face workshop will last 2 days and facilitate real-time discussion, review, and modifications of the Delphi statements, a technique used in previous literature [33]. This workshop will include presentations by the research team on the findings from the 2 previous Delphi rounds and breakout sessions whereby stakeholders from a particular expertise will be grouped together. In groups they will be asked to (1) consider the phrasing and interpretation of the statements on the digital tools that they are experts in, (2) develop recommendations for further consideration when developing digital tools for a health-related matter, (3) discuss any changes to statements with consensus lower than 70%, and (4) help compose the final set of statements.

Following this review and finalization of the statements, participants will again be presented with a 7-point Likert scale for each statement with the following options: strongly disagree, disagree, somewhat disagree, either agree or disagree, somewhat agree, and agree.

Statements that meet the appropriate level of consensus will be included in a final list of recommendations for countries when developing, implementing, and maintaining digital health strategies during a health emergency or pandemic.

It should be noted that only participants who took part in rounds 1 and 2 will be eligible to take part in the face-to-face workshop. That is because experts who took part in both rounds will have a clear understanding of the statements being asked, improving the efficiency of the workshop as well as the discussion. Those who do not take part in both rounds 1 and 2, or are unavailable for the face-to-face discussion, will still be given the opportunity to rate the statements online through Qualtrics through an invitation link by email.

**Figure 3:**
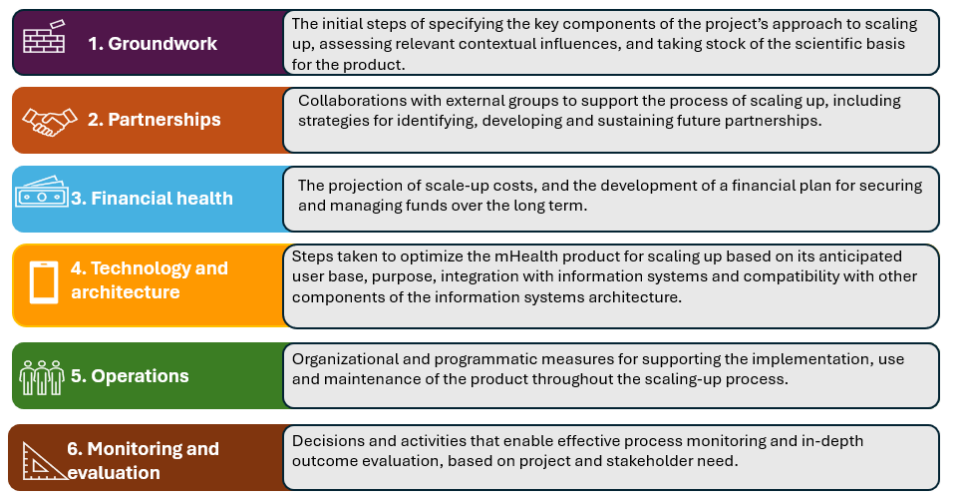
mHealth (mobile health) assessment and planning for scale toolkit-axes of scale taken from the World Health Organization mHealth Assessment and Planning for Scale toolkit.

In addition, the panel will be asked to complete, in their expertise group, the MAPS toolkit assessment on the digital tool on which they are knowledgeable. The MAPS toolkit will enable the panel members to assess their digital tool from a sustainable perspective from six distinct, yet complementary axes: (1) groundwork, (2) partnerships, (3) financial health, (4) technology and architecture, (5) operations, and (6) monitoring and evaluation (Figure 3).

The MAPS toolkit works by participants responding to the self-assessment questions [14]. There are 2 suggested approaches to achieve this, either through an individual assessment or team assessment. For this modified Delphi study, participants will be grouped according to expertise and required to complete a team assessment of the digital tool which they knowledgeable on. It is recommended that the toolkit should take approximately 1.5-2 hours to complete in its entirety. The research team has allotted 3 hours for completion on day 1 of the workshop.

After the participants complete the MAPS Toolkit, the self-assessment questions scores will be calculated on 3 levels, the overall score (total score combining all axes), the axis scores (a separate score for each of the 6 axes of scale), and the domain scores (specific scores for the domains within each axis of scale).

The scoring procedures will enable the research team to explain the overall progress through the scaling up process, as well as their internal strengths and weaknesses in quantitative terms. The ability to compare ratings across axes and domains will assist the research team to identify which areas require additional improvement [14].

#### Data Analysis

Descriptive statistics will be used to summarize and analyze the data from Delphi rounds 2 and 3. This data includes characteristics of the participants, the number of participants who took part during each round, along with their demographic details, the median and mode for each item, an indication of the current level of consensus (based in the IQR), the summary of participant responses as to why they have ranked statements, on the 7-point Likert scale, in the way they did; and descriptive statistics (proportion or mean and SD where appropriate) will be used to summarize the individual responses to each statement in the Delphi round. The quantitative data, if appropriate, will be entered into SPSS for analysis and all experts’ opinions will be weighted equally.

Descriptive statistics will be undertaken on the entire dataset after entering the data to provide a percentage of the overall response to each question. The median and IQR will be reported for each item. In addition, to investigate the qualitative data (open-ended questions), we will use rapid qualitative analysis. Statements that are the same or very similar will be amalgamated, and all comments will be grouped thematically.

Statements that reach consensus will be categorized according to the digital tool in which the statement relates to and will be mapped to one of the following concepts: development, implementation, maintenance, or impact. We will also subcategorize the statements according to the MAPS toolkit domains and axes. Furthermore, a figure will be designed that presents the flow of items through the Delphi process and presents the final ratings of statements for each of the 4 aforementioned concepts.

### Subgroup Analysis

A subgroup analysis will be conducted if feasible on respondents’ profession, highest degree or education, and gender. For this study, gender refers to “socially constructed and enacted roles and behaviors which occur in a historical and cultural context and vary across societies and over time” [34].

This is to identify what statements are more likely to be endorsed based on a person’s demographic data and to identify variances in justifications for rating statements.

### Ethical Considerations

This study was reviewed and approved by the National Committee on Research in the Social Sciences and Humanities [NO. P.12/23/825] in Malawi. This study will conform to the Declaration of Helsinki [35].

Informed consent in writing from participants will be required before data collection starts. The investigators will support the participants to withdraw from the study at any stage of the research process if they wish to do so without giving reasons. Participants will be reminded that involvement in the study is entirely voluntary. By participating in this Delphi study, participants are contributing to important research on digital surveillance systems in Malawi. We do not anticipate any negative outcomes or risks from participating in this study. We do not intend to cause any distress to stakeholders, as this Delphi study will ask about their own experiences with these systems.

Individual rating of the statements for each round will be anonymous to the other participants. However, participants are identifiable to the research team. Participants are required to provide a full name at the start of each round, this is to allow the research team to follow up with incomplete or not started survey forms. This is stated in the participant information leaflet. It should be noted that the research team will deidentify any information provided after round 3 and before the final publication. No statements or ratings will be traceable back to the participants.

In addition, for the experts who are eligible and available to take part in the round 3 face-to-face workshop, support for travel costs will be provided. The amount of compensation for travel costs will be determined based on fuel expenses to and from the venue in Malawi.

## Results

This study was reviewed and approved by the National Committee on Research in the Social Sciences and Humanities [NO. P.12/23/825] in Malawi. Round 1 invitations were sent on February 8, 2024. Round 2 invitations were sent on May 13, 2024. Data collection is ongoing.

## Discussion

### Expected Outcomes

This protocol describes a process to gather consensus and recommendations from leading experts in Malawi on digital tools deployed during the COVID-19 pandemic. This is an area of limited research and thus, comparison. However, we anticipate that the modified Delphi approach through open-ended round 1 questions and the option to participate in an in-person workshop in round 3 will allow for greater in-depth exploration of the digital tools deployed and improve the quality of this work.

We believe that the study findings will provide meaningful insights into stakeholders’ perceptions of the effectiveness of these digital tools. In addition, this study will facilitate the development of recommendations on the development, implementation and maintenance of digital tools for future disease outbreaks. This study will also provide valuable insights and feedback on the digital tools through the application of the World Health Organization MAPS toolkit. The findings will inform implementers and foreign donors on digital health development initiatives and will help countries increase their disease surveillance capacity in the future.

### Limitations

We foresee that a limitation will be the number of experts engaged in this research, the anticipated decline in response rate and incomplete responses. There will be 40 experts in total, which is approximately 13 for each digital tool. However, we hope that the ability to conduct the research online will make the questions and statements accessible to the experts and boost response rate. Furthermore, this consensus study focuses on the perspectives of the developers of the digital tools and does not incorporate the user experience of the digital tools.

### Conclusions

In summary, global health epidemics, pandemics, and endemics can happen unexpectedly and therefore, there is a need for governments to ensure preparedness to tackle such occurrences. This research will encapsulate digital tool development and impact and facilitate the development of recommendations for future digital surveillance strategies.

## Appendix

Multimedia Appendix 1 Mapping and matching digital health tools to strengthen Malawi’s COVID-19 response. Table adapted from (Digital Health Systems to support pandemic response in Malawi - Squarespace 2021).

## Abbreviations

EOC: emergency operations center
LMIC: low- and middle-income country
MAPS: mHealth Assessment and Planning for Scale
mHealth: mobile health
OHSP: One Health Surveillance Platform
