# Supplementary material for: Evaluation of the Development, Implementation, Maintenance, and Impact of 3 Digital Surveillance Tools Deployed in Malawi During the COVID-19 Pandemic: Protocol for a Modified Delphi Expert Consensus Study
Source: JMIR Res Protoc. 2024 Dec 31;13:e58389. doi: 10.2196/58389 (PMC11733520; doi:10.2196/58389)
Supplement: Multimedia Appendix 1 [file resprot_v13i1e58389_app1.pdf]

Supplementary Table 1: Mapping and matching digital health tools to strengthen Malawi's COVID-19 response. Table adapted from (Digital Health Systems to support pandemic response in Malawi - Squarespace 2021)

Blue illustrates digital health tools supporting Malawi's COVID-19 response. The Y-axis are the digital tools deployed for COVID-19. The X-axis details the tools' functionality.

	Case Management	Contact tracing	Coordination and operations	Diagnostic tools	Event-based Surveillance (including rapid response teams, case investigators)	Health facility and provider administration	Infection prevention and control	Laboratory systems	Learning and training	One Health	Points of entry	Risk communication and community engagement	Routine surveillance	Supply chain	Vaccine and planning
AirConnect/Applet															
Chipatal Cha pa Foni (Viamo's 3-2-1 service															

CommCare															
Community Health Toolkit															
Digital Community Scorecard App															
eIDSR Case Based Surveillance App Malawi															
Emergency Operations Center Call Center Exchange															
HealthConnect															
Laboratory Information Management System															
Leap															
Malawi's HMIS (DHIS2 with Tracker and Android)															

Ministry of Health Covid-19 Dashboard															
Ministry of Health WhatsApp Chatbot & USSD application (RapidPro)															
One Health Surveillance Platform (DHIS2)															
Open LMIS															
Organized Network Services for Everyone's Health (ONSE)															
Remote Training for Frontline Health Workers															
Swoop Aero															
U-Report															

World Continuing Education Alliance															
